# Auto-correlations of microscopic density fluctuations for Yukawa fluids in the generalized hydrodynamics framework with viscoelastic effects

**DOI:** 10.1038/s41598-022-26401-w

**Published:** 2022-12-19

**Authors:** Ankit Dhaka, P. V. Subhash, P. Bandyopadhyay, A. Sen

**Affiliations:** 1 Institute for Plasma Research, Bhat, Gandhinagar, Gujarat 382428 India; 2 Homi Bhabha National Institute, Training School Complex, Anushaktinagar, Mumbai, 400094 India; 3 ITER-India, Institute for Plasma Research, Bhat, Gandhinagar, Gujarat 382428 India

**Keywords:** Physics, Plasma physics

## Abstract

The present work develops a theoretical procedure for obtaining transport coefficients of Yukawa systems from density fluctuations. The dynamics of Yukawa systems are described in the framework of the generalized hydrodynamic (GH) model that incorporates strong coupling and visco-elastic memory effects by using an exponentially decaying memory function in time. A hydrodynamic matrix for such a system is exactly derived and then used to obtain an analytic expression for the density autocorrelation function (DAF)—a marker of the time dynamics of density fluctuations. The present approach is validated against a DAF obtained from numerical data of Molecular Dynamics (MD) simulations of a dusty plasma system that is a practical example of a Yukawa system. The MD results and analytic expressions derived from the model equations are then used to obtain various transport coefficients and the latter are compared with values available in the literature from other models. The influence of strong coupling and visco-elastic effects on the transport parameters are discussed. Finally, the utility of our calculations for obtaining reliable estimates of transport coefficients from experimentally determined DAF is pointed out.

## Introduction

A Yukawa system generally consists of an ensemble of a large number of charged particles embedded in an electrically neutral or quasi-neutral medium such that the bare charge of a particle is shielded by the medium particles. Yukawa systems have attracted a lot of research interest because of their importance in many fields including space physics^1^, astrophysical systems^2^, gas discharges^3,4^, microelectronics, colloidal systems, the edge of thermonuclear fusion systems^5–7^, condensed matter physics (specially for understanding the phase transitions^8–10^ in 2D and 3D systems), etc. Such systems are also extensively studied in laboratories to investigate various fundamental physics problems associated with many body systems^11–13^. As a large amount of information is already available in the literature regarding the domain of existence and the fundamental importance of Yukawa systems, further details about them are omitted here. Good overviews of their basic properties, applications, and methods of experimental and theoretical studies of Yukawa systems can be found in several books and review papers^14–17^. Complex plasmas or dusty plasmas are a particular class of Yukawa systems where nano-meter to micro-meter sized charged particles (called dust) are suspended in a partially ionized plasma. Many past studies have investigated transport processes, crystallization, phase transitions and collective modes in Yukawa systems using various approaches such as Molecular Dynamics (MD) simulations^18,19^, a Generalized Hydrodynamics (GH) model^20^, a Quasi-Localized Charge Approximation (QLCA)^21^ and Kinetic Theory^22^ etc.

In a Yukawa system the inter-particle shielded potential between the embedded grains is taken to be of the form:

1 $$\begin{aligned} \phi (r) = \frac{Q^2}{4\pi \varepsilon _0}\frac{\exp (-r/\lambda _D)}{r}, \end{aligned}$$

where *r* is the separation between two particles having charge *Q*, $\varepsilon _0$ is the permittivity of free space and $\lambda _D$ is the screening length arising from the background plasma. Yukawa systems can be characterized by two dimensionless parameters, namely, the Coulomb coupling strength defined as $\Gamma = Q^2/(4\pi \varepsilon _0a_{ws}k_BT_D)$ and the screening strength defined as $\kappa = a_{ws}/\lambda _D$ where $a_{ws}$ is the average inter-particle distance, $T_D$ is the temperature and $k_B$ is Boltzmann’s constant. The Coulomb coupling parameter and screening strength can be adjusted to achieve longer or shorter correlations among the particles, that can characterize the phase state of the system as being a fluid or a solid^23^.

The time evolution of small density fluctuations of fluids around the equilibrium values can be used to understand the transport process at a fundamental level. This was famously noted way back by Landau-Placzek^24^, who had observed that the variation of density fluctuations in time can be described by linear hydrodynamic equations of irreversible thermodynamics. A similar statement by Kubo that “the linear response of a given system to an external perturbation is expressed in terms of fluctuation properties of the system in thermal equilibrium” is also noteworthy^25^. One way to understand the time evolution of fluctuations is to write down conservation laws such as conservation of density, momentum, and energy in the hydrodynamic limit with quantities having small fluctuations around their equilibrium values. After linearising the equations one can further simplify them using thermodynamic relations to reach a set of coupled equations. This set of equations relates fluctuations of density, momentum, and energy to their equilibrium values. The system of equations when written in matrix form has a coefficient matrix, which is normally called the hydrodynamic matrix^26^. Following some reasonable assumptions, these equations can be solved for variation of density fluctuations in time in terms of various equilibrium values. To understand the time dynamics of density fluctuations, a time auto-correlation function of density fluctuations can be constructed. This is found to yield much important information on transport processes in fluids. Such a calculation for the case of ‘simple fluids’ can be found in Ref.^26^. This observation has been implemented in the light scattering studies from ideal mono-atomic liquids by Mountain^27^, to construct the generalized structure factor and other dynamical quantities. Following the work of Mountain, the same approach has been used to study thermodynamic density fluctuations for a dense charged fluid (a strongly coupled one component plasma (OCP)) by Vieillefosse and Hansen^28^. They added a local electric field term in the momentum equation to incorporate the effects of charged particles. This procedure to understand transport parameters is more accurate as one starts from an unambiguous quantity, the density fluctuations, and is valid for complicated situations like materials with non-pairwise potentials such as warm dense matter etc^29^. Recent studies of OCPs have also shown the estimation of dynamic structure factor in various screening regimes using an alternate approach known as the method of moments^30,31^.

The situation in Yukawa fluids is more interesting as there exists a third possibility to obtain DAF through experiments. Dusty plasmas, a particular class of Yukawa fluids, are extensively studied in the laboratory and their dynamics are captured in the form of particle trajectories using high speed camera systems^3,32–35^. To exploit this aspect we need to have an accurate expression for DAF derived from a proper hydrodynamic matrix for Yukawa systems. It can be noted from a comparison between Refs.^28,29^ that the DAFs, hence the transport parameters, are different for simple fluids and OCP because of the additional term in the momentum equation. In Yukawa fluids, strong coupling effects as well as visco-elastic effects (sometimes called memory effects) need to be incorporated in the fluid equations. The Generalized Hydrodynamics model (GH) is one such model that incorporates both these features^36^. The GH model potentially bridges the gap between hydrodynamic and kinetic regimes by extending the usual Navier–Stokes model to higher wavelength-frequency domains. As a result, this model is applicable over a large extent of correlations as compared to other hydrodynamic models. This model has been applied to a dusty plasma system by Kaw and Sen^20^ for studying low-frequency dust acoustic modes.

Recent advances in Molecular Dynamic (MD) simulations give another dimension to this method. Using such simulations, we can numerically calculate the density fluctuations and hence the Density Autocorrelation Function (DAF). This numerically constructed DAF can then be compared with theoretical DAF to obtain important transport parameters and acoustic speeds. Recently Cheng and Frenkel^29^ used this combination to successfully calculate transport parameters of simple fluids as well as for warm dense matter. These past studies of density fluctuations of simple fluids using an analytical form of the DAF have proved very useful for understanding many fundamental physics issues and in practical applications. A primary goal of our present study is to derive a similar analytical form of the DAF for a complex system, such as a Yukawa system, whose dynamical characteristics are significantly different from that of a simple fluid. Such an analytical form of the DAF has not been derived so far for a Yukawa system although Yukawa systems have been extensively studied for a long time. Just as in the case of the DAF for a simple fluid, our present result can be used to gain insights into the transport properties of complex systems like a dusty plasma which is well represented by a Yukawa model. Furthermore, a dusty plasma system offers a convenient means of carrying out an experimental verification of the results obtained using our derived DAF. This has also served as a major motivation for our present work.

In our work, we have used the generalised hydrodynamic model to represent the complex system and derived an appropriate hydrodynamic matrix and the density auto-correlation function. The analytic form of the DAF incorporates contributions from strong coupling effects such as visco-elasticity and enables us to study its impact on various physical parameters such as sound speed and transport coefficients. Our analytic results are further validated against MD simulations by fitting the expression of DAF to numerical results. As the DAF expression contains transport coefficients, the comparison with numerical results also provide estimations of transport coefficients.

## Hydrodynamic matrix and DAF from generalized hydrodynamic model

### Hydrodynamic matrix

Assuming the hydrodynamic regime, the conservation laws for number density $\rho ({\varvec{r}},t)$ and energy density $e({\varvec{r}},t)$ can be written as

2 $$\begin{aligned}{} & {} m\frac{\partial }{\partial t}\rho ({\varvec{r}},t) + {\varvec{\nabla }}\cdot {\varvec{p}}({\varvec{r}},t) = 0, \end{aligned}$$

3 $$\begin{aligned}{} & {} \frac{\partial }{\partial t}e({\varvec{r}},t) + {\varvec{\nabla }}\cdot {\varvec{J}}^e({\varvec{r}},t) = 0, \end{aligned}$$

where ${\varvec{J}}^e({\varvec{r}},t)$ is the energy current density and ${\varvec{p}}({\varvec{r}},t)$ is the momentum current density. Now, assuming the local deviation in number density $\delta \rho ({\varvec{r}},t)$ to be small, Eq. (2) can be linearised as

$$\begin{aligned} {\varvec{p}}({\varvec{r}},t) = m[\rho +\delta \rho ({\varvec{r}},t)]{\varvec{u}}({\varvec{r}},t)\approx m\rho {\varvec{u}}({\varvec{r}},t) \equiv m{\varvec{j}}({\varvec{r}},t), \end{aligned}$$

with *m* as mass, ${\varvec{u}}({\varvec{r}},t)$ as velocity and ${\varvec{j}}({\varvec{r}},t)$ as the local density current. Using the above approximation, the continuity Eq. (2) can be rewritten in the form

4 $$\begin{aligned} \frac{\partial }{\partial t}\delta \rho ({\varvec{r}},t) + {\varvec{\nabla }}\cdot {\varvec{j}}({\varvec{r}},t) = 0. \end{aligned}$$

Considering the heat continuity Eq. (3), the heat current is defined as

$$\begin{aligned} {\varvec{J}}^e({\varvec{r}},t) = (e+P){\varvec{u}}({\varvec{r}},t) - \lambda \nabla T({\varvec{r}},t), \end{aligned}$$

where $e=U/V$ is the equilibrium energy density, $\lambda$ is thermal conductivity and *P* is the overall pressure. Using the expression for $J^e$, the *energy equation* (3) can be rewritten as,

5 $$\begin{aligned} \frac{\partial }{\partial t} \delta \underbrace{ \left( e({\varvec{r}},t) - \frac{e+P}{\rho }\rho ({\varvec{r}},t) \right) }_{\text {Density of Heat Energy : }q({\varvec{r}},t)} - \lambda \nabla ^2\delta T({\varvec{r}},t) = 0. \end{aligned}$$

$\delta q({\varvec{r}},t)$ is related to $\delta \rho ({\varvec{r}},t)$ and $\delta T({\varvec{r}},t)$ as,

6 $$\begin{aligned} \delta q({\varvec{r}},t) = T\delta s({\varvec{r}},t) ={} & {} \frac{T}{V}\frac{\partial S}{\partial \rho }\delta \rho ({\varvec{r}},t)+ \frac{T}{V}\frac{\partial S}{\partial T}\delta T({\varvec{r}},t) = -\frac{T\beta _v}{\rho }\delta \rho ({\varvec{r}},t)+\rho c_v \delta T({\varvec{r}},t), \end{aligned}$$

where $\beta _v = \left( \frac{\partial P}{\partial T}\right) _\rho = -\rho \left( \frac{\partial (S/V)}{\partial \rho }\right) _T$ is the thermal pressure coefficient. Using Eqs. (4) and (6), the energy continuity equation (5) can be rewritten as

7 $$\begin{aligned} \left( \frac{\partial }{\partial t} -\frac{\lambda }{\rho c_v}\nabla ^2 \right) \delta T({\varvec{r}},t) + \frac{T\beta _v}{\rho ^2 c_v}{\varvec{\nabla }}\cdot {\varvec{j}}({\varvec{r}},t) = 0. \end{aligned}$$

The use of equilibrium thermodynamic relations in Eq. (6) is justified on the same grounds as the use of irreversible hydrodynamic equations to describe the time evolution of reversible microscopic fluctuations^24–26^. In other words, the irreversibility is at the macroscopic scale of the transport processes but there exists reversibility at the local microscopic scale of the fluctuations.

As discussed earlier, a Generalized Hydrodynamic model^20^ is used for linear momentum equation. This model is a generalization of hydrodynamic equations of motion by taking into account the Maxwell’s relaxation theory. The GH model uses a non-local viscoelastic operator with an exponentially decaying memory function of relaxation time $\tau _m$ known as the viscoelastic relaxation time. Theoretical formalism to generalise the hydrodynamic equations and applications of the GH model are delineated in Refs. ^37,38^. The linear momentum equation from Generalized Hydrodynamic model^20^ can be written as

8 $$\begin{aligned}{} & {} \left( 1+\tau _m \frac{\partial }{\partial t} \right) \left[ \frac{\partial }{\partial t}{\varvec{j}}({\varvec{r}},t) + \frac{1}{m}\nabla P({\varvec{r}},t) + \frac{Q\rho }{m}\nabla \phi \right] -\frac{\eta }{\rho m } \nabla ^2 {\varvec{j}}({\varvec{r}},t) -\frac{\eta /3 +\zeta }{\rho m}\nabla {\varvec{\nabla }} \cdot {\varvec{j}}({\varvec{r}},t)=0, \end{aligned}$$

with $\eta$ as shear viscosity and $\zeta$ as the bulk viscosity. $\tau _m$ is the relaxation time of the memory function which is modeled as an exponentially decaying function in time^20^. Fluctuations in $P({\varvec{r}},t)$ to first order in $\delta \rho ({\varvec{r}},t)$ and $\delta T({\varvec{r}},t)$ are related as

9 $$\begin{aligned} \delta P ({\varvec{r}},t) = \frac{1}{\chi _T \rho }\delta \rho ({\varvec{r}},t)+\beta _v\delta T({\varvec{r}},t), \end{aligned}$$

where $\chi _T$ is isothermal compressiblity. Using Eq. (9), the momentum, energy and continuity equation, for a Yukawa system, can be rewritten as

10 $$\begin{aligned}{} & {} \left( 1+\tau _m \frac{\partial }{\partial t} \right) \left[ \frac{\delta \rho ({\varvec{r}},t)}{m\chi _T \rho } +\frac{\beta _v}{m}\delta T({\varvec{r}},t) +\frac{Q\rho }{m}\nabla \phi \right] +\left\{ \left( 1+\tau _m \frac{\partial }{\partial t} \right) \frac{\partial }{\partial t} -\frac{\eta }{\rho m } \nabla ^2 - \frac{\eta /3 +\zeta }{\rho m}\nabla {\varvec{\nabla }} \cdot \right\} {\varvec{j}}({\varvec{r}},t)=0 \end{aligned}$$

11 $$\begin{aligned}{} & {} \left( \frac{\partial }{\partial t} - \frac{\lambda }{\rho c_v}\nabla ^2 \right) \delta T({\varvec{r}},t) + \frac{T\beta _v}{\rho ^2 c_v}{\varvec{\nabla }}\cdot {\varvec{j}}({\varvec{r}},t) = 0, \end{aligned}$$

12 $$\begin{aligned}{} & {} \frac{\partial }{\partial t}\delta \rho ({\varvec{r}},t) + {\varvec{\nabla }}\cdot {\varvec{j}}({\varvec{r}},t) = 0. \end{aligned}$$

The relation between density $\rho$ and $\phi$ can be established by using a modified Helmholtz^39^ like equation that is a static version of Eq. (3) of Ref.^40^.

13 $$\begin{aligned} (\nabla ^2 -\lambda _D^{-2})\phi = 4\pi Q \delta \rho . \end{aligned}$$

This equation relates the potential induced due to variation in the charge density ($Q\delta \rho$) for the systems interacting via Yukawa interaction. Now, the GH momentum equation (Eq. (10)) along with particle and energy conservation laws (Eqs. (11), (12)) can be transformed using a double transform with respect to space (Fourier) and time (Laplace) to obtain a relation of density ${\tilde{\rho }}({\varvec{k}},s)$, particle current density $\tilde{{\varvec{j}}}({\varvec{k}},s)$ and local temperature ${\tilde{T}}({\varvec{k}},s)$ with their corresponding Fourier components, $\rho _k$, $T_k$ and ${\varvec{j}}_k$, at $t=$0. The Laplace transform of function *f*(*t*) has the form ${\mathscr {L}}\{f(t)\} = \int \exp (\iota s t)f(t)dt$. Assuming ${\varvec{k}}$ to be in the *z* direction (without losing generality) and neglecting electromagnetic effects, the longitudinal part of Eqs. (10), (11) and (12) can be written in (*k*, *s*) space. These system of equations can be written in matrix form with $b=\frac{4\eta /3 +\zeta }{\rho m}$ and $a = \frac{\lambda }{\rho c_v}$ as follows.

14 $$\begin{aligned}{} & {} \underbrace{\left[ \begin{array}{ccc} -\iota s &{} 0 &{} \iota k \\ 0 &{} -\iota s + ak^2&{} \frac{T\beta _v \iota k}{\rho ^2 c_v} \\ \frac{\iota {\varvec{k}}{\tilde{\rho }}_{{\varvec{k}}}(s)}{1-\iota s \tau _m}\left[ \frac{1}{m\chi _T \rho } +\frac{\omega _p^2}{k^2+\lambda _D^{-2}}\right] &{} \frac{\iota {\varvec{k}} \beta _v(1-a\tau _mk^3)}{m (1-\iota s \tau _m)} &{} \begin{array}{c} \frac{1}{(1-\iota s \tau _m)}\left( \left[ \frac{1}{m\chi _T \rho } + \frac{\beta _v^2}{\rho ^2 m c_v} \right] k^2\tau _m\right) \\ +\frac{bk^2 -(\iota s+\tau _ms^2) }{(1-\iota s \tau _m)}\\ \end{array} \end{array}\right] }_{\mathrm{Hydrodynamic Matrix : }H_L(s,k)} \left[ \begin{array}{c} {\tilde{\rho }}_k(s) \\ {\tilde{T}}_k(s) \\ {\tilde{j}}^z_k(s) \\ \end{array}\right] \nonumber \\{} & {} \quad = \left[ \begin{array}{c} \rho (0) \\ T (0) \\ {j}^z_k (0) + \frac{\tau _m \dot{j_{kz}}(0)}{1-\iota s \tau _m}\\ \end{array}\right] . \end{aligned}$$

The coefficient matrix in Eq. (14) is called the Hydrodynamics matrix $H_L(s,k)$.

### Density autocorrelation function (DAF)

The dispersion relation for the longitudinal collective modes is determined by the poles of the inverse of $H_L(s,k)$ i.e. the roots of Eq. (15).

15 $$\begin{aligned} \det {H_L(s,k)} = 0. \end{aligned}$$

Assuming $-\iota s = z$, $\det {H_L(s,k)}$ can be written as follows

16 $$\begin{aligned} \det {H_L(s,k)}{} & {} =z^4\tau _m + z^3(1+a\tau _mk^2)+ z^2k^2(a+b+\tau _m K_T)\nonumber \\{} & {} \quad + zk^2\left[ ak^2\left( b+\frac{K_T\tau _m}{\gamma } \right) +\frac{\omega _p^2}{k^2+\lambda _D^{-2}}+K_T\right] \nonumber \\{} & {} \quad + ak^4\left( \frac{K_T}{\gamma }+\frac{\omega _p^2}{k^2+\lambda _D^{-2}} \right) , \end{aligned}$$

where definitions of $K_T$ as $K_T=\frac{\gamma }{\rho m \chi _T}$ and thermodynamic relation $c_p = c_v +\frac{T\chi _T \beta _v^2}{\rho }$ have been used.

The approximate roots of Eq. (15) of the order $k^2$ can be determined using power series method as shown in Appendix 1, as follows.

$$\begin{aligned} z_1{} & {} = -\underbrace{a \left( 1 -\alpha \right) }_{A}k^2 \text { where } \alpha = \left( \frac{K_T(\gamma -1)/\gamma }{K_T+\frac{\omega _p^2}{k^2+\lambda _D^{-2}}} \right) , \\ z_{2\pm }{} & {} =\pm \iota \underbrace{\sqrt{K_T + \frac{\omega _p^2}{k^2+\lambda _D^{-2}}}}_{c_s}k - \underbrace{\frac{1}{2}\left( b+a\alpha -\frac{\omega _p^2\tau _m}{k^2+\lambda _D^{-2}} \right) }_{\Gamma _s}k^2,\\ z_4{} & {} = -\frac{1}{\tau _m} + \left[ b-\frac{\omega _p^2 \tau _m}{k^2+\lambda _D^{-2}}\right] k^2, \end{aligned}$$

where the fluctuations in temperature and density are instantaneously uncorrelated^26^ ($\left\langle T_k \rho _k\right\rangle =0$) and ${\varvec{k}}$ can be chosen to ensure $j_k^z=0$. Considering these simplifications, Eq. (14) can be solved for ${\tilde{\rho }}_{{\varvec{k}}}(s)$.

17 $$\begin{aligned} \frac{{\tilde{\rho }}_{k}(s)}{\rho _{k}}={} & {} \frac{z^3\tau _m +z^2(1+a\tau _mk^2+zk^2(a+b+\tau _m K_T))+(\gamma -1)K_Tk^2/\gamma }{\tau _m (z-z_1)(z-z_{2+})(z-z_{2-})(z-z_4)}. \end{aligned}$$

Using the roots of $\det H_L(s,k)=0$, we can solve for ${\tilde{\rho }}_k$ by finding partial fraction coefficients corresponding to each root. As we show later, the coefficient corresponding to the fourth root in the density autocorrelation will be zero so the same is excluded from here onwards.

18 $$\begin{aligned} \frac{{\tilde{\rho }}_{k}(s)}{\rho _{k}} ={} & {} \frac{\alpha }{z-z_1}+\frac{1-\alpha }{2}\left( \frac{1}{z-z_{2+}} + \frac{1}{z-z_{2-}}\right) . \end{aligned}$$

Now, above equation can be written as following using an inverse transform followed by a multiplication of $\rho _{-k}(0)$ on both sides and thermal averaging.

19 $$\begin{aligned} \left\langle \rho _k (t) \rho _{-k}(0) \right\rangle =\alpha \exp \left( -Ak^2t\right) +\left( 1-\alpha \right) \exp (-\Gamma _sk^2t)\cos (c_skt). \end{aligned}$$

The attenuation constant $\Gamma _s$, coefficient *A* and acoustic speed $c_s$ are given by

20 $$\begin{aligned} \Gamma _s ={} & {} \frac{1}{2}\left( b+a\alpha -\frac{\omega _p^2\tau _m}{k^2+\lambda _D^{-2}} \right) , \end{aligned}$$

21 $$\begin{aligned} A ={} & {} a(1-\alpha ), \end{aligned}$$

22 $$\begin{aligned} c_s ={} & {} \sqrt{K_T + \frac{\omega _p^2}{k^2+\lambda _D^{-2}}}. \end{aligned}$$

It can be noted immediately that putting t = 0 in Eq. (19) reduces DAF to unity as expected, which in turn needs the coefficient of the fourth root to be zero.

Equation (19) contains two terms, the first one is a diffusive term driven by thermal diffusion and the second term is a damping cosine. The frequency of the cosine is described by sound speed, and the decay rate is determined through attenuation constant $\Gamma _s$, hence called sound attenuation constant. The coefficients defined in Eqs. (20)–(22) are modified by viscoelastic memory effects. Indeed, in the asymptotic limit of $\lambda _D \rightarrow \infty$ and $\tau _m \rightarrow 0$, Eq. (19) reduces to the density autocorrelation function of a classical one-component plasma (without memory effects) as given in Vieillefosse and Hansen^28^.

## Validation with MD simulations

### Calculation of DAF through MD

The DAF described in Eq. (19) can be independently calculated through the first principle method using molecular dynamics (MD) simulations. The MD simulations numerically solve the coupled equations of motion of particles for a given inter-atomic force field. As the solver progresses in time, the dynamical evolution of the system is recorded by storing the positions and velocities of particles, also known as trajectories. This in turn produces a full 6N + 1 dimensional phase space of the system with N being the number of particles. The physical observables can now be calculated from this data with various statistical tools.

In the present study we have performed MD simulations of N = 131,072 point-like particles using a well benchmarked MD code LAMMPS^41^ using the inter-particle Yukawa potential described by Eq. (1) and the simulation parameters are tabulated in Table 1. The particle number N has been chosen by considering value of $k_{min}a_{ws} = \sqrt{4\pi /N}$ as per the $O(k^2)$ assumption in the theoretical model. A periodic boundary condition is implemented in each dimension to minimize the finite size effects.

The system is initially equilibrated using a thermostating procedure^42^ followed by a NVE production run. The thermostatting procedure is important as it removes the sensitivity of the results to initial conditions of the simulation. This also prevents to a large extent the propagation of numerical errors that are sensitive to initial conditions. The NVE production run of 60,000 $\omega _{pd}t$ time steps has been used for storing particle trajectories. The $\omega _{pd}$ here is the dust plasma frequency given by $\omega _{pd}=\sqrt{nQ^2/m\varepsilon _02a_{ws}}$.

A simulation duration of 60,000 $\omega _{pd}t$ is chosen to obtain a sufficiently large number of ensembles such that the DAFs do not differ when a simulation with an even longer duration is performed. The lengths and times are normalized with $a_{ws}$ and $2\pi \omega _{pd}^{-1}$ respectively. All other quantities are also normalized using the normalization scheme employed for time and space. The use of normalised quantities makes the calculations independent of a particular physical system or a system of units. The normalisation also helps one to decide a suitable time step *dt* of a simulation so that the solver converges and the repeatability of results is ensured. Once the results are obtained one can simply use the expression of $\omega _p$ to convert the parameters to any particular system of units as required. As the potential given in Eq. (1) falls as *r* increases, a potential truncation radius is used to speed up the computation which is chosen as per a benchmarked criterion explained by Liu and Goree^43^.

**Table 1:** List of simulation parameters in normalised units.

S.no.	Parameter	Values
1.	Particle number	131,072
2.	Equilibration steps	$5000\omega _pt$
3.	Production steps	$60,000\omega _pt$
4.	Step size *dt*	$0.005\omega _pt$
5.	$\Gamma$	[5, 200]
6.	$\kappa$	[1, 3.5]

In order to calculate DAF from particle trajectories, the microscopic particle density in the reciprocal space for N point particles with positions ${\varvec{r}}_i(t)$ is defined as

23 $$\begin{aligned} {\rho }_{{\varvec{k}}}(t) = \frac{1}{V}\int _V \sum _{i=0}^N\delta ({\varvec{r}}-{\varvec{r}}_i(t))\exp (\iota {\varvec{k}}\cdot {\varvec{r}})d{\varvec{r}}. \end{aligned}$$

The reciprocal space vector ${\varvec{k}}$ is related to system dimensions $(L_x,L_y,L_z)$ as ${\varvec{k}} = \{ 2\pi n_x/L_x,2\pi n_y/L_y,2\pi n_z/L_z \}$. The minimum value of magnitude of reciprocal space vector $|{\varvec{k}}|_{min}=9.79\times 10^{-3}$. Now, the microscopic particle density ${\rho }_{{\varvec{k}}}(t)$ is transformed with a Fast Fourier Transform (FFT) in time and Wiener–Khintchine^44^ theorem is used to calculate density autocorrelation in time using following equation.

24 $$\begin{aligned} \left\langle \rho _k (t) \rho _{-k}(0) \right\rangle = {\mathscr {F}}^{-1} \{ {\tilde{\rho }}_k(\omega )^{\dagger }{\tilde{\rho }}_k(\omega )\}. \end{aligned}$$

**Figure 1:**
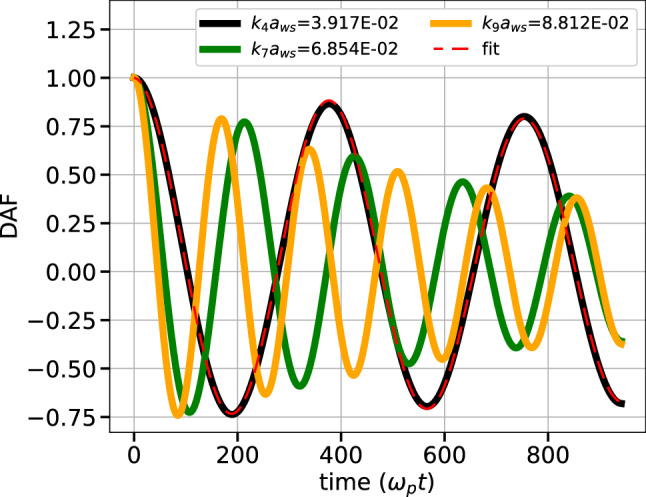
DAF curves generated using MD simulations (solid lines) and curve obtained by fitting MD data with Eq. (19) (broken lines) for $\Gamma$ = 60 and $\kappa$ = 2.

The DAF calculated from the simulation for $\Gamma$ = 60 and $\kappa$ = 2.0 is shown in Fig. 1 (solid lines) for 3 different modes. Eq. (19) is then best fitted to this curve using non-linear least square fits resulting in optimal parameters for each mode. A fitted curve for $k_4$ mode is also shown in Fig. 1 (dashed lines) which closely follows the DAF from MD. Additional figures of DAFs for different simulation parameters are available in Supplementary Material.

### Comparison with MD and discussion

In the previous subsection, the DAF generated through MD data is found to fit well with Eq. (19). This fitting has been performed by fixing the various transport parameters such as $c_s$, $\Gamma _s$, $\gamma$ and thermal diffusivity $a = \lambda /\rho c_p$ as fitting parameters. As the fitting procedure involves multiple parameters, it should be noted that not all of them can be varied arbitrarily. Firstly, the parameter $c_s$ explicitly depends only on the frequency of the DAF time series and hence gets decoupled from the others. The quantity *A* independently appears in the exponential of the first term and it also appears in the exponential of the second term (corresponds to the viscosity constant). So these two terms (*A* and the viscosity term) cannot be arbitrarily chosen to fit the MD data.

**Figure 2:**
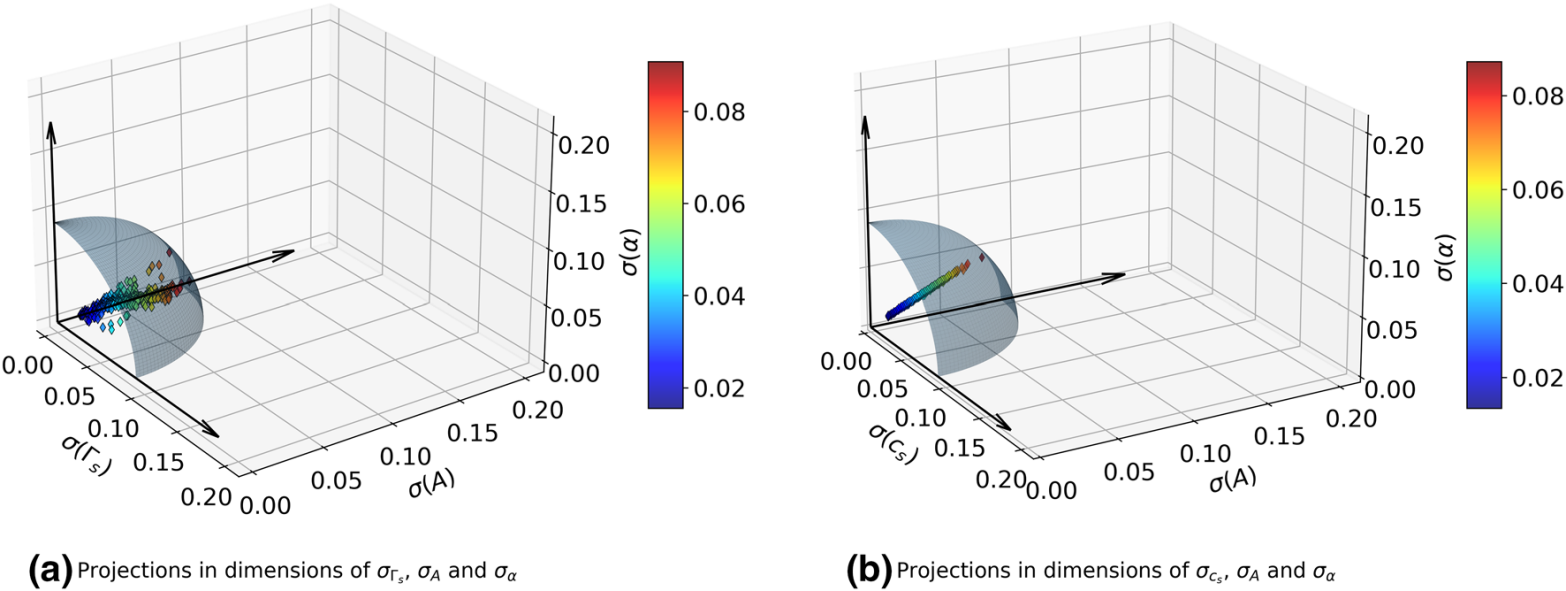
3D projections of MSDs from the parameter hyperspace in normalized units. The colors of the points represents the Euclidean norms and shaded spherical surface encloses the region where $\sigma < \sigma _{max}$.

In order to have better confidence in the fitting procedure, an exercise involving the statistical uncertainties is also performed. A multidimensional space for the statistical errors around the fitted parameters is constructed. The statistical errors are quantified in terms of mean squared deviations (MSD) of each parameter. As we have four fitting parameters for each *k*, the number of dimensions of this space will be four. These values are independently calculated for each *k* and the maximum MSDs for individual parameters are collected. The spread in the unit of fractions (ratio of the error to value of the parameter) are denoted as $\pm \sigma$ with $\sigma _{max} = [0.0003, 0.049, 0.077, 0.041]$ corresponding to $[c_s, A, \Gamma _s, \alpha ]$. Here $\alpha$ is the coefficient of the first term in Eq. (19). To visualize the extent of deviations, the MSDs are shown in 3D projections of the four-dimensional hyperspace of parameters in Fig. 2 for all modes. The Fig. 2a shows the populations of MSDs estimated in the parametric space of $\sigma _{\Gamma _s}$, $\sigma _A$ and $\sigma _{\alpha }$ dimensions. Similar information is shown in Fig. 2b corresponding to $\sigma _{c_s}$, $\sigma _A$ and $\sigma _{\alpha }$ dimensions. The colors of each scatter point show the Euclidean norm of the point which conveys the maximum possible deviation of all dimensions combined. It is evident from the figures that the populations of MSDs are limited to a small region within the parametric hyperspace, hence the fitting procedure is statistically accurate.

In addition to ensuring the statistical accuracy of the fitting procedure we have also carried out an independent check on the validity of the estimated transport coefficients by comparing them with values available through various models in the literature. The comparison presented below covers Yukawa systems in 2D and 3D along with a discussion on the physical effects of strong coupling terms and the memory effects on the transport parameters.

Before going to a one-by-one comparison, it is important to check the reduction of Eq. (19) in some important asymptotic limits. For an ideal uncharged fluid ($\omega _p =0$ or $\kappa \rightarrow \infty$), Eq. (19) exactly reduces to the DAF of an ideal fluid^26^ as shown below

25 $$\begin{aligned} \left\langle \rho _k (t) \rho _{-k}(0) \right\rangle ={} & {} \left( \frac{\gamma -1}{\gamma } \right) \exp (-D_Tk^2t)+\frac{1}{\gamma }\exp (-\Gamma _{SF}k^2t)\cos (c_skt), \end{aligned}$$

where

$$\begin{aligned} \Gamma _{SF} = \frac{1}{2}\left( b+a\frac{\gamma -1}{\gamma }\right) \text {and } D_T = a/\gamma . \end{aligned}$$

A comparison between Eqs. (19) and (25) shows that the transport terms such as $c_s$, $\gamma$ and thermal conductivity are modified through a new form of compressibility. While the longitudinal viscosity appearing in $\Gamma _s$ is modified through a term containing the relaxation time $\tau _m$.

The speed of acoustic modes^45^ in Yukawa systems can be estimated using various methods and models including molecular dynamic (MD) simulations^46^, QLCA^47^, and fluid models supplemented with an equation of state, etc. For estimations of the adiabatic constant, parametric equation of state obtained from MD simulations or other models, are used in some reported cases^48,49^. Among all these methods the QLCA approach requires high coupling regimes for charges to be localized^21^ and the fluid approach is reported to be accurate in $\kappa \le 3$ regimes^50^. Also, a direct experimental implementation is difficult for all the above cases.

**Figure 3:**
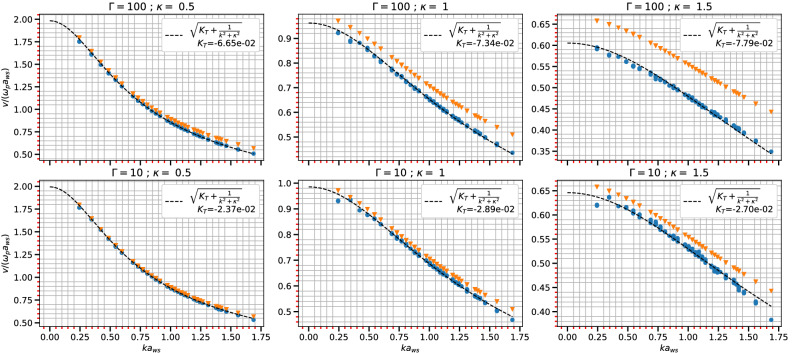
Sound speed ($c_s$) with $ka_{ws}$ for different $\Gamma$ and $\kappa$ in a 3D system. The inverted triangles represent $\sqrt{1/(k+\kappa ^2)}$ term and dashed line shows the fitting with analytic expression for sound speed from Eq. (22).

The following important point related to the expression for sound speed using the QLCA method by Kalman et al.^51^ is worth noting here. According to Eq. (19) of Ref.^51^ the approximate expression of longitudinal phase velocities of Yukawa Systems for the limit of $k \rightarrow 0$ is given as

26 $$\begin{aligned} s_L^2 = \omega _p^2a_{ws}\left[ f(\kappa ) + \frac{1}{\kappa ^2}\right] , \end{aligned}$$

with $f(\kappa )$ as a fitting function. The expression obtained through present derivation as in Eq. (22) is also in a similar form but with an explicit *k* dependence and a more physically meaningful *k* independent term, compressibility $K_T$. Thus the present form avoids the need for ambiguous parametric fitting on the estimation of sound speed. A similar form of dispersion relation is also reported in other places, for example, see Ref. ^20^ and references therein.

**Figure 4:**
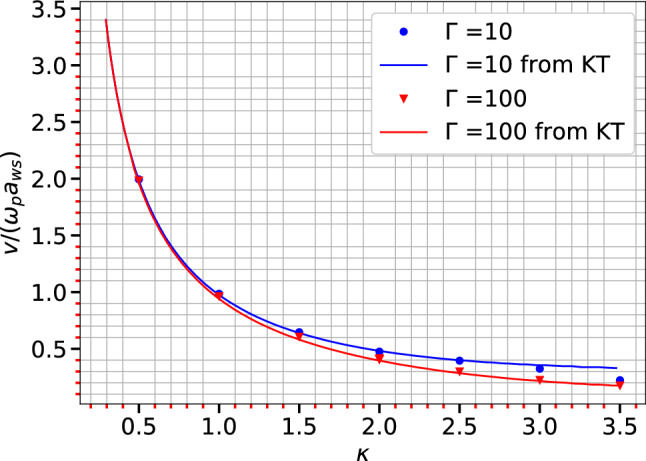
Comparison of sound speed obtained in $k\rightarrow 0$ limit for a 3D system with results from Khrapak and Thomas^48^.

Now the left hand side of Eq. (22) can be obtained from fitting Eq. (19) with MD data for each wave-vector *k*. These values can be further fitted with the expression in right hand side of Eq. (22) as shown in Fig. 3. The fitting procedure is also capable of separating the wavelength dependent term from the other term in expression. The circles in the Fig. 3 show MD point for the left hand side of Eq. (22) and broken lines show the fit using the expression in the right hand side. The term $\sqrt{1/(k+\kappa ^2)}$ is shown with inverted triangles and calculated values of $K_T$ is also mapped in Fig. 3. The values for $c_s$ are then extrapolated to $k\rightarrow 0$ and compared with results of Khrapak and Thomas^48^ in Fig. 4. The figure shows the comparison for two values of $\Gamma$s and different values of $\kappa$ ranging from 0.5 to 3.5 for a 3D system. The acoustic speed estimation for $\kappa$ = 3.5 and $\Gamma =$ 10 shows a slight deviation from the results of Khrapak and Thomas. The work by Khrapak and Thomas uses a parametric form of the equation of state to derive the acoustic speed. This approximation is not strictly valid beyond $\kappa$ = 3, and this could be the cause of the deviation of their result from that of our present study beyond $\kappa$ = 3.

**Figure 5:**
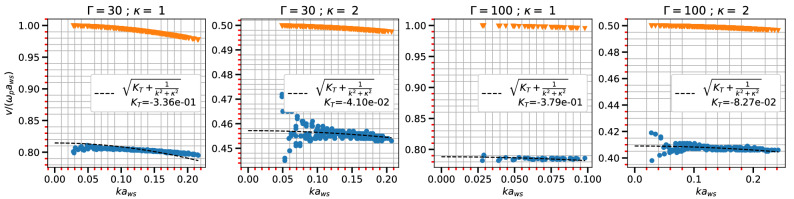
Sound speed ($c_s$) with $ka_{ws}$ for different $\Gamma$ and $\kappa$ in a 2D system. The inverted triangles represent $\sqrt{1/(k+\kappa ^2)}$ and dashed line shows the fitting with analytic expression for sound speed from Eq. (22).

**Figure 6:**
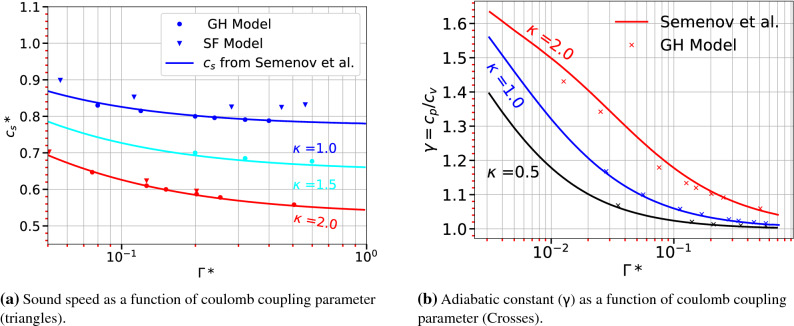
Comparison of sound speed and adiabatic constant across with results from Semenov et al.^50^ (solid lines). Following the same normalization used in Ref. ^50^, the sound speed is normalized as $c_s* = c_s\kappa /(\omega _{pd}a_{ws})$ and effective coulomb coupling parameter as $\Gamma *=\Gamma /\Gamma _m$ where $\Gamma _m(\kappa )=131/[1-0.388\kappa ^2+0.138\kappa ^3-0.0138\kappa ^4]$.

To check the validity of the present model for 2D dusty plasma, the following comparisons are performed. A plot for 2D cases exactly similar to Fig. 3 is shown in Fig. 5. Similarly, the sound speed estimated for 2D cases and a comparison of present calculations with Semenov et al.^50^ is shown in Fig. 6a. The solid lines are from Ref.^50^ and circles are from the present calculations using a combination of MD data, Eqs. (19) and (22). The inverted triangles are for the limiting case of simple fluids as in Eq. (25). Both the axes are normalized as described in the caption to make it analogous to the work of Semenov et al.^50^. The comparisons are presented for three cases with $\kappa$ = 0.5, 1, 2 and for many values of $\Gamma$ from 1 to 100. Following important points can be noted from Fig. 6a. Firstly, the present model agrees well with the results of Semenov et al.^50^. Secondly, as $\kappa$ increases the values calculated with the GH model (Eq. 19) approach the values estimated using the simple fluid model. As discussed earlier this point is also in line with expectations. This in turn validates the present derivation. A similar comparison for adiabatic constant $\gamma$ is also given in Fig. 6b. It should be noted that for calculating $\gamma$, an equivalence between the quantity $(\gamma - 1)/\gamma K_T$ for the cases with and without background, as explained by Salin^52^, is used. Here also the present method can estimate values closer to that of Semenov et al.^50^ even for $\gamma$s close to one.

From the above discussions, it is clear that the sound speeds and γs obtained using the present model and MD data in rigorous ways agree with other available models in the literature even though they are very different from the present approach. For completeness, in the rest of this section a comparison of another important transport parameter, the thermal conductivity is presented.

The thermal conductivity estimation can be done by equilibrium MD simulations using Green–Kubo formula, which is based upon the fluctuation–dissipation theorem^53^. This method involves the computation of heat current auto-correlation which has a slow convergence^54^. The definition of local heat current is not unique^29,54^, and in many cases (for example, if the potential is not pairwise additive) accurate estimation of thermal conductivity requires specific formulation in GK method^29,54,55^. Another method to calculate thermal conductivity is to use non-equilibrium molecular dynamics (NEMD) simulations^56^ by inducing a local temperature gradient in a small region of the system to estimate the heat flux^54^. In principle NEMD methods closely mimic the experimental situations and are not difficult to implement in MD, but there exist many computational issues^54^. The limitations include finite-size effects and non-linear responses due to temperature gradient^57^. A review of both equilibrium methods and non-equilibrium methods with merits and demerits are available in a study by Schelling et al.^54^. However, both of the aforementioned methods are difficult to deploy in experimental Yukawa systems. For GK methods, experimentally estimating the local heat current is challenging, while in non-equilibrium methods creating a local thermal gradient and keeping the overall system at a constant average temperature, and measuring local heat flux is difficult.

**Figure 7:**
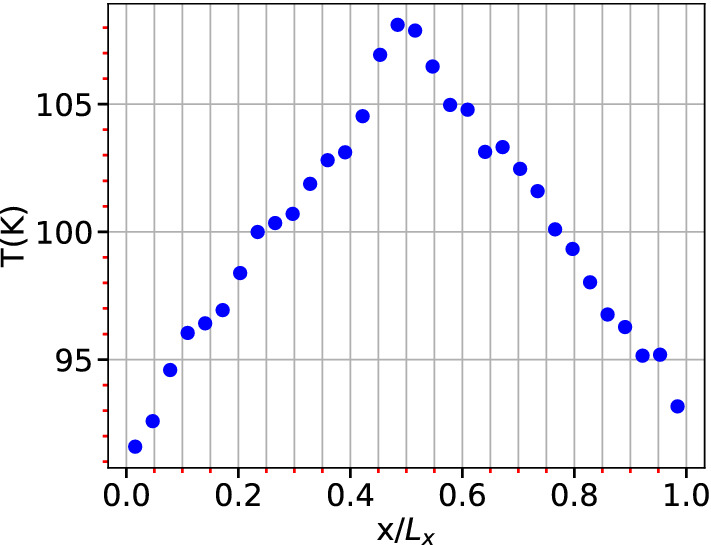
Temperature profile constructed for estimation of thermal conductivity from NEMD method.

To validate the prediction of thermal conductivity using the present model, MD calculations for NEMD are separately performed as described below. For NEMD calculation, a reversible non-equilibrium method proposed by Müller–Plathe^56^ (MP) is used. It is based on the idea of deliberately imposing a heat flux and measuring the system response as a temperature gradient profile. The system is divided into 32 slabs along ${\hat{x}}$ direction and heat flux is imposed by exchanging the kinetic energy of the ‘coldest’ particle in one slab with ‘hottest’ in another slab. The induced temperature gradient, as the response of the system, is measured by taking ensemble averages. The temperature profile after establishing the temperature gradient is shown in Fig. 7. Now, for a 2D system the thermal conductivity is related to heat flux using Fourier’s law as

27 $$\begin{aligned} \lambda = \frac{E}{2Lt \left\langle \delta T/\delta x\right\rangle }, \end{aligned}$$

where E is the total energy exchanged in time *t*, *L* is length of slab and $\left\langle \delta T/\delta x \right\rangle$ is the ensemble average of temperature gradient. The mean values of the gradients of temperature ($\Delta T/\Delta x$) were calculated by considering the interval $x/L_x \in (0,0.5)$ and $x/L_x \in (0.5,1)$.

**Figure 8:**
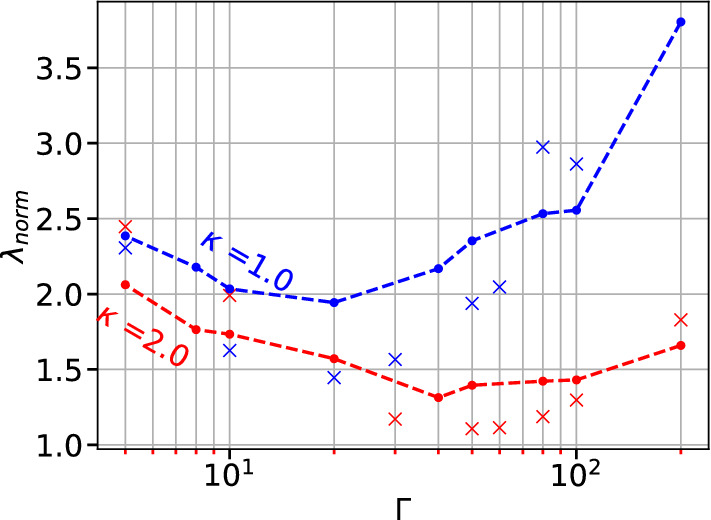
Comparison of heat conductivity calculated with NEMD Method (dashed lines), and the present work using Eq. (19). The thermal conductivity is normalized with $\omega _p$ as $\lambda _{norm} = {\lambda }/{nk_{\beta }\omega _pa_{ws}^2}$.

Eq. (27) is used to estimate the thermal conductivity from a known heat flux and temperature profile. A comparison of the results obtained using Eq. (19) with that obtained using NEMD for $\kappa =1$ and $\kappa =2$ for many values of $\Gamma$ is shown in Fig. 8. The present simulation agrees well with NEMD method considering the reported inaccuracy of NEMD method up to 20%.

As discussed earlier, the NEMD method has its own computational disadvantages. As the present method closely follows the analytical treatment and the DAF is calculated from particle fluctuations, it is free from such problems but can be prone to statistical errors that arise from fitting procedures.

Validation of the final parameter in Eq. (19) namely $\Gamma _s$ is not performed here as the same expressed in the form of Eq. (20) is not available in literature.

An important extension of this work could be an analytical estimation of the stress autocorrelation function (SACF) which is related to the viscosity using a Green–Kubo formula^58^. In this regard, the matrix equation (Eq. 14) can be extended by incorporating the transverse currents ($j_x$,$j_y$). This would result in a hydrodynamic matrix of order 5, which can be inverted to approximately solve the system of equations resulting in analytical expressions of current densities. These current densities can be used along with the conservation law of momentum to calculate the stress tensor.

One could also have an alternate approach to obtain viscosity using the auto correlation of the time derivative of the current density. For example, this can be achieved using the following form of expression, which has been previously used for the case of simple fluids^26^.

28 $$\begin{aligned} \eta = \frac{\beta m^2}{V}\lim _{\omega \rightarrow 0}\lim _{k\rightarrow 0} Re \int _0^\infty \frac{1}{k^2} \langle {\varvec{{\dot{j}}}}^x_k(t) {\varvec{{\dot{j}}}}^x_{-k}(0)\rangle \exp (\iota \omega t)dt. \end{aligned}$$

Furthermore, we would like to make the following important point. As shown above, the present approach of using Eq. (19) and MD simulations can be used for accurate estimation of various transport parameters in a single framework. Moving forward, as explained below, there exists an interesting possibility to replace MD data with experimental data. One of the beauties of laboratory dusty plasma systems is their simplicity in obtaining the particle trajectories using fast cameras^3,10,35^. These particle trajectories can be used to obtain a DAF. The experimentally obtained DAF can then be matched with Eq. (25) as discussed earlier. In other words, the particle trajectories obtained through MD simulation in the present work can be replaced with experimental measurements. As discussed earlier, the experimental implementation of previous individual models for each thermodynamic quantities such as $c_s$, $\gamma$, $\lambda$ are difficult, need more complicated diagnostics and more importantly, require separate treatments. Using Eq. (19) of the present work with experimentally measured particle trajectories enables the estimation of many important transport parameters in a single framework. For normal systems like simple fluids, this cross-validation is not possible as experimentally measuring the individual particle dynamics and fluctuations is impossible. In short, the present work opens up a window to cross-validate the dynamics of microscopic fluctuations at hydrodynamic limits with theoretical, computational, and experimental means. An experimental attempt for the same is presently under way and will be reported later.

## Summary

In the present work, an analytical relation for the time dynamics of DAF for a Yukawa fluid has been explicitly derived in a generalized hydrodynamic framework which is valid over large spatial correlations and incorporates viscoelastic effects. This analytical form is then used directly for the estimation of various transport coefficients. This analytical form can also be compared directly with experimental or MD data to obtain important transport coefficients using proper fitting procedures. A potential generalization of the present work is to extend the calculations by including transverse current density components to obtain useful analytical expressions for other important parameters like the stress-autocorrelation function. Furthermore, the expression of the DAF as in Eq. (19) can be usefully employed to estimate an upper limit of integration of the various GK formulae, when the DAF is obtained as finite time series from experimental data.

### Supplementary Information

Supplementary Information.

## Data Availability

The datasets used and/or analysed during the current study available from the corresponding author on reasonable request.

## Appendix

### Method of solving Eq. (15)

The roots of a quartic equation such as Eq. (16) of the form

29 $$\begin{aligned} P(k)z^4 +Q(k)z^3 +R(k)z^2 +S(k)z^1+T(k) = 0, \end{aligned}$$

with the coefficients P, Q, R, S and T being functions of *k*, can be approximately estimated using a power series method. In this method, a trial solution of the form $z_t = z_{t0} +z_{t1}k+z_{t2}k^2...$ is substituted in equation. Then the terms with same order of *k* are collected together and the coefficient $z_{t0}$ is estimated by considering the lowest order terms in *k*. For Eq. (16), the lowest order coefficient of trial solution is

30 $$\begin{aligned} z_{t0}= 0,0,0,-1/\tau _m. \end{aligned}$$

Now, this process is repeated again with substitution of trial solution with the calculated $z_{t0}$ from previous step to estimate next order coefficient i.e. $z_1$. For each value of $z_{t0}$, the order (k) coefficient of trail solution is

31 $$\begin{aligned} z_{t1} = 0,\pm \sqrt{K_T+\frac{\omega _p}{k^2+\lambda _D^{-2}}},0. \end{aligned}$$

This is repeated until required approximation in order of *k*, which is $O(k^2)$ in this paper, is reached. The second order coefficient of the trial solution is

32 $$\begin{aligned}{} & {} z_{t2} =-a ( 1 -\alpha ),-\frac{1}{2}\left( b+a\alpha -\frac{\omega _p^2\tau _m}{k^2+\lambda _D^{-2}} \right) ,\left[ b-\frac{\omega _p^2 \tau _m}{k^2+\lambda _D^{-2}}\right] . \end{aligned}$$

The final approximate roots of order $O(k^2)$ are then estimated from Eqs. ((30)–(32)) as shown in “Hydrodynamic matrix and DAF from generalized hydrodynamic model” section.
