# Supplementary material for: Auto-correlations of microscopic density fluctuations for Yukawa fluids in the generalized hydrodynamics framework with viscoelastic effects
Source: Sci Rep. 2022 Dec 19;12:21883. doi: 10.1038/s41598-022-26401-w (PMC9763351; doi:10.1038/s41598-022-26401-w)
Supplement: Supplementary file 1 — Supplementary Information. [file 41598_2022_26401_MOESM1_ESM.pdf]

Supplementary Materials

Equations in Laplace-Fourier Space

An important step for analytically deriving the functional form of density autocorrelation function (DAF) is to solve the system of equations constructed using conservation laws. Firstly, the GH momentum equation (Eq. 10) along with particle and energy conservation laws (Eq. 11 & Eq. 12) are transformed. The spatial part of the equations is transformed using a Fourier transform and a Laplace transform is used with respect to time. This will result in relations of number denisty $\tilde{\rho}(\boldsymbol{k},s)$, particle current density $\tilde{\boldsymbol{j}}(\boldsymbol{k},s)$ and local temperature $\tilde{T}(\boldsymbol{k},s)$ with their corresponding Fourier components, ρ_k, T_k and $\boldsymbol{j}_k$, at t =0.

Using the form of Laplace transform discussed in manuscript, the longitudinal part of Eqs. (10), (11) and (12) can be written in (k,s) can be obtained as follows.

$$-\imath s\tilde{\rho}_k(s)+\imath k\cdot\tilde{j}_{kz}(s)=\rho_k(0)$$

$$(-\imath s+ak^2)\tilde{T}_k(s)+\frac{T\beta_v}{\rho^2 c_v}\imath k\tilde{j}_{kz}(s)=T_{\boldsymbol{k}}(0)$$

$$\frac{\imath k\tilde{\rho}_k(s)}{1-\imath s\tau_m}\left[\frac{1}{m\chi_T\rho}+\frac{\omega_p^2}{k^2+\lambda_D^{-2}}\right]+\frac{\imath k\beta_v(1-a\tau_m k^3)}{m(1-\imath s\tau_m)}\tilde{T}_{\boldsymbol{k}}(s)+\qquad\frac{\tilde{j}_{kz}(s)}{1-\imath s\tau_m}\left(\left[\frac{1}{m\chi_T\rho}+\frac{\beta_v^2}{\rho^2 mc_v}\right]k^2\tau_m+bk^2-(\imath s+\tau_m s^2)\right)$$

$$=j_{kz}(0)+\frac{\tau_m\dot{j}_{kz}(0)}{1-\imath s\tau_m}$$

where $b=\frac{4\eta/3+\zeta}{\rho m}$ and $a=\frac{\lambda}{\rho c_v}$. The above system of equations can be written in the matrix form where the coefficient matrix is the Hydrodynamic matrix for Yukawa system in GH framework.

Additional Plots of DAFs

We have performed a large number of simulations for different coupling and screening parameters of a Yukawa system in order to cover a wide span of parameter space and thereby verify our model. In order to show the accuracy of the model we have represented the errors as the 3D plots in parameter hyperspace. Apart from this, the results obtained are compared with values from different models to verify the accuracy of the study. Additionally, an useful demonstration of the model would be to provide findings similar to Fig 1 of manuscript for various parameters. As there exists a large number of modes (k) spanned in each dimension, it would result in a large number of DAF curves. Since it is not possible to show all the plots for each DAF, we have plotted a few cases for each simulation for reference.

Figure 1 below represents the plots of DAF for cases of screening parameter κ=2 and coupling parameter Γ =10,30,50,60,80,200. The analytic function fitted on top of numerical result is shown for one wave-vector using red broken lines and three other modes of DAF are plotted for clarity. Similarly, Figure 2 represents the plots of DAF for screeining parameter $\kappa=1$ and coupling parameter $\Gamma=10,30,50,60,80,100$. These plots along with the errors represented using 3D plots in parameter hyperspace for each mode k help us to conclude that the model is accurate across a range of parameter regime.

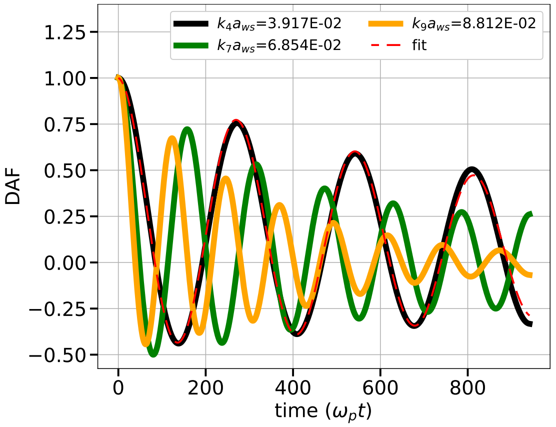

(a) Γ=10

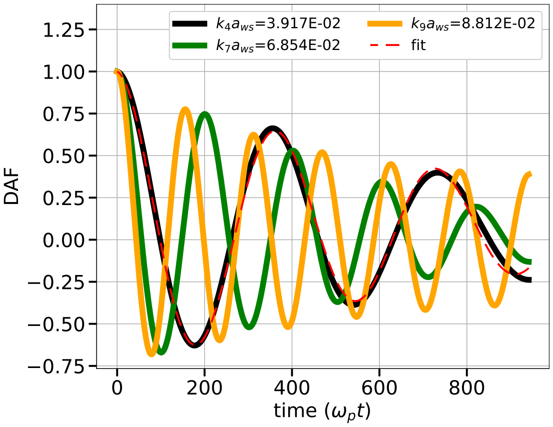

(b) Γ=30

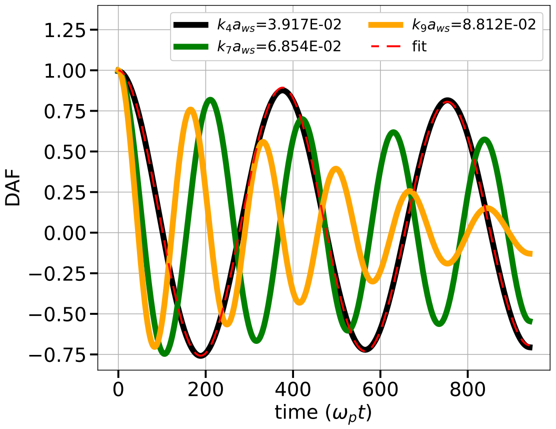

(c) Γ=50

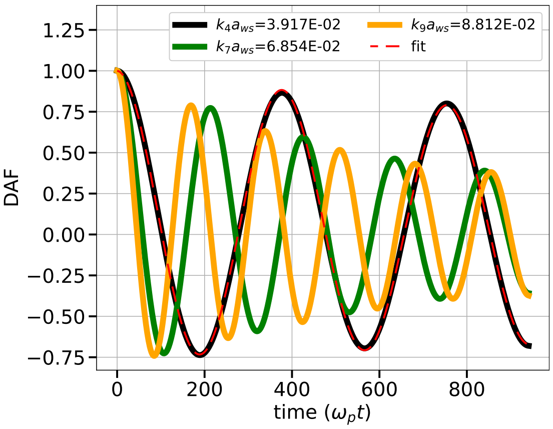

(d) Γ=60

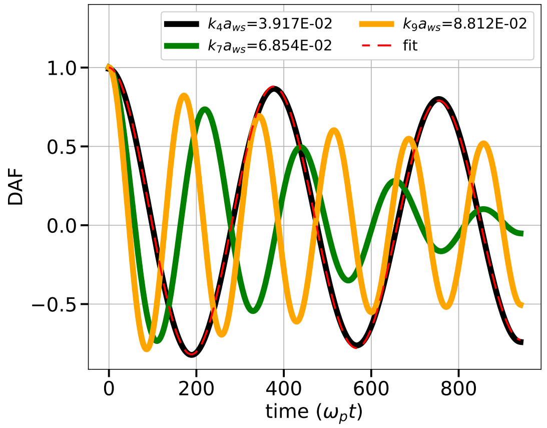

(e) Γ=80

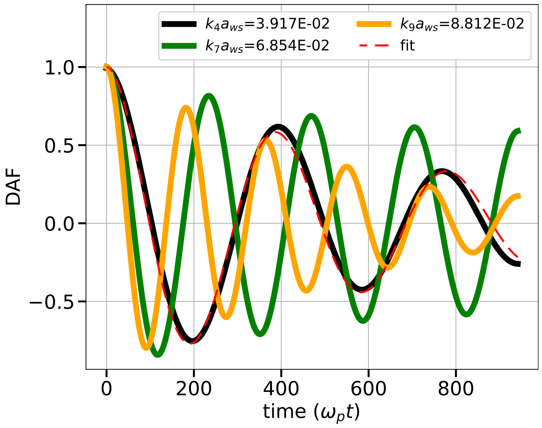

(f) Γ=200

Figure 1. DAF curves generated using MD simulations (solid lines) and curve obtained by fitting MD data with Eq. (19) (broken lines) for κ=2

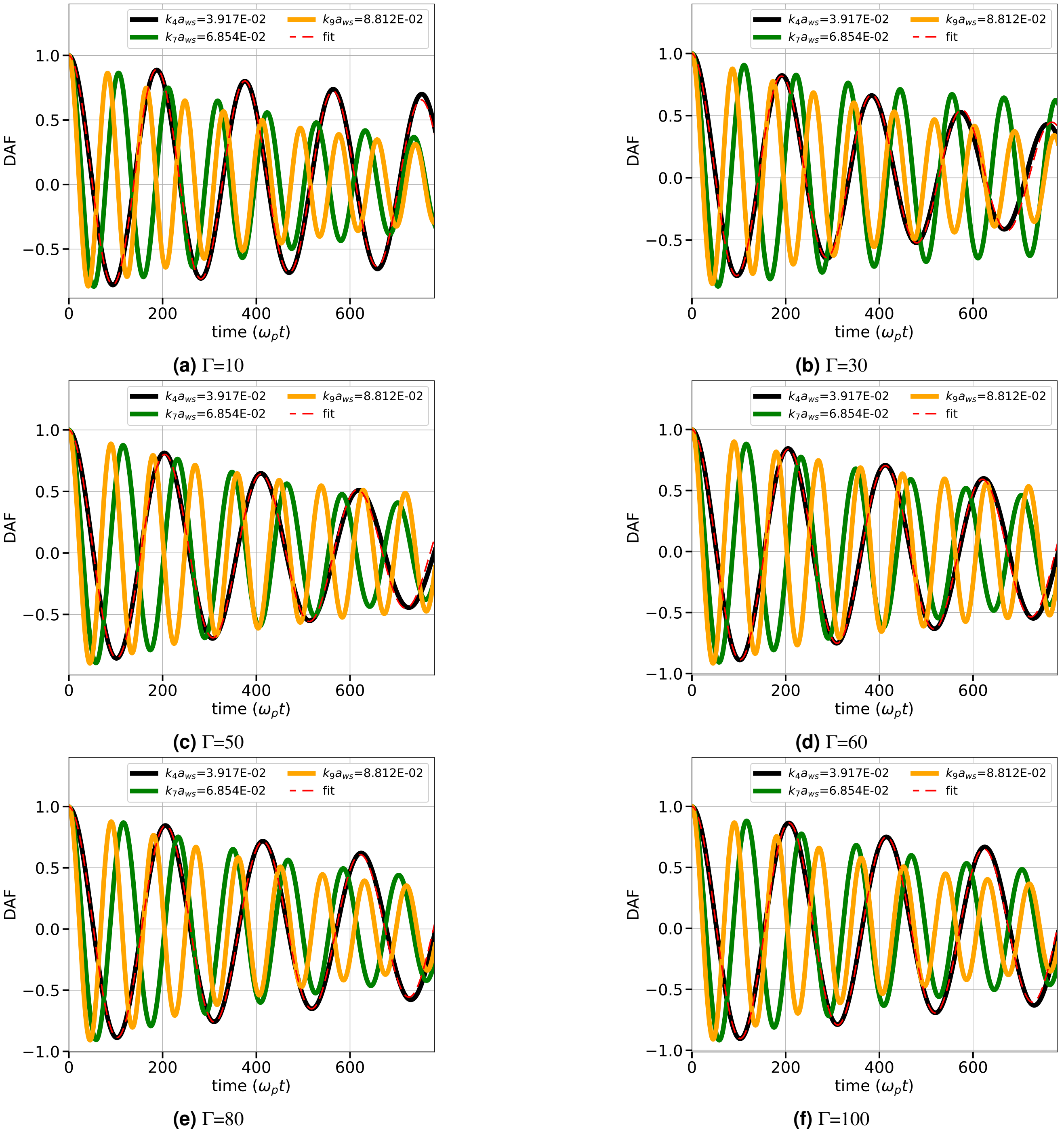

(a) Γ=10 **(b)** Γ=30 **(c)** Γ=50 **(d)** Γ=60 **(e)** Γ=80 **(f)** Γ=100

Figure 2. DAF curves generated using MD simulations (solid lines) and curve obtained by fitting MD data with Eq. (19) (broken lines) for κ=1
